# Culturally Adapted Manual Assisted Problem-Solving Intervention for Older Adults With Suicidal Ideation (E-CMAP): A Study Protocol From Pakistan

**DOI:** 10.1192/bjo.2024.127

**Published:** 2024-08-01

**Authors:** Sehrish Tofique, Nasim Chaudhry, Imran Bashir Chaudhry, Jahanara Miah, Nusrat Husain

**Affiliations:** 1 Pakistan Institute of Living and Learning, Karachi, Pakistan; 2 Ziauddin Univeristy, Karachi, Pakistan; 3 University of Manchester, Manchester, United Kingdom; 4 University of Manchester, Manchester, United Kingdom

## Abstract

**Aims:**

Suicide poses a significant public health issue, and the presence of suicidal thoughts stands out as a prominent risk factor, highlighting the importance of addressing this aspect for early intervention and prevention efforts. While older adults face an elevated risk of attempted suicide, research in this domain is currently constrained. This study aims to enhance and evaluate the efficacy of an E-CMAP (Culturally Manual Assisted psychological intervention for Elderly) in mitigating suicidal ideation among individuals aged 55 years and older in Pakistan.

**Methods:**

The study will be carried out in 2 phases. Phase 1 is cultural adaptation and refinement of the intervention and phase 2 is exploratory randomised control trial. In Phase 1, focus groups were conducted (N = 2) with Health professionals and service users and carers for adaptation of CMAP manual for suicidal ideation. In Phase 2 randomized exploratory trial will be conducted with 192 older adults with suicidal ideation randomized either to 1) E-CMAP added to Treatment As Usual (TAU) or TAU arm. ECMAP is a problem solving intervention comprising 6 sessions delivered one to one over 3 months by trained therapists. All participants will be assessed at baseline and after intervention (i.e. 3 months) for suicidal ideation, hopelessness, depression, health-related quality of life, coping resources, satisfaction with intervention, and episodes of self-harm.

**Results:**

Thematic Analysis of focus group discussions indicates that participants expressed a preference for incorporating a religious element into distraction techniques, delivering information about the significance of medical treatment, showcasing recorded sessions illustrating problem-solving techniques, and involving family throughout the intervention period.

**Conclusion:**

A culturally tailored psychosocial intervention that incorporates problem-solving and cognitive components has the potential to decrease the risk of suicide among older adults.

